# Diffusion Bonding of 1420 Al–Li Alloy Assisted by Pure Aluminum Foil as Interlayer

**DOI:** 10.3390/ma13051103

**Published:** 2020-03-02

**Authors:** Fan Wu, Wei Chen, Bing Zhao, Hongliang Hou, Wenlong Zhou, Zhiqiang Li

**Affiliations:** 1 Power Beam Processing Laboratory, AVIC Manufacturing Technology Institute, Beijing 100024, China; werner_nju@163.com; 2 Beijing Aeronautical Manufacturing Technology Research Institute, Beijing 100024, China; zhao6833@163.com (B.Z.); hou_hl@163.com (H.H.); 3 School of Materials Science and Engineering, Dalian University of Technology, Dalian 116024, China; zhouwenlong1965@163.com

**Keywords:** diffusion bonding, interface microstructure, shear strength, interlayer

## Abstract

The Al–Li alloy is becoming popular for aerospace application owing to their low density, high specific strength, good corrosion resistance, etc. The diffusion bonding/superplastic forming (DB/SPF) structure of titanium alloy has been widely used in the aerospace industry. In order to broaden the application of Al–Li alloy, it is necessary to develop its diffusion bonding and superplastic forming (DB/SPF) technology. In the present study, diffusion bonding of 1420 Al–Li alloy assisted by pure aluminum foil was conducted on Gleeble-3500 thermal simulation system under different bonding parameters, the results show that the bonding temperatures have direct influence on the interface microstructure and bond strength of joints. Meanwhile, when the pure aluminum interlayer was introduced into the diffusion bonding process, the alloying element diffusion across the bond can improve the interface integrity and the mechanical properties. The joint formation mechanism with interlayer was investigated in detail, the development and application of this method was explored.

## 1. Introduction

The Al–Li alloy has become a promising member of aerospace used aluminum alloys due to its low density, high specific strength, good corrosion resistance, and excellent superplastic forming performance [1,2,3]. In recent years, the multilayer lightweight structural components obtained by the superplastic forming/diffusion bonding (SPF/DB) technology has been widely used in the aerospace industry [4,5,6]. Therefore, the development of DB/SPF technology for the Al–Li alloy is of great significance to further recommend the integration and lightweight of aircraft components and reduce flight costs. As the Al–Li alloy is easy to obtain microstructure with equiaxed fine grains and show excellent superplasticity, the key to develop the SPF/DB technology of Al–Li alloy is to obtain a high quality diffusion bonding joint.

Diffusion bonding (DB) is a solid-state welding process in which two prepared surface are joined at a certain range of temperature under a certain pressure and holding for a certain time [7,8,9]. As is known to all, diffusion bonding of the aluminum alloy is always a tough task due to the tenacious oxide film covered on the substrate which act as diffusion barrier and prevent the formation of high quality bond joint [10]. There is general agreement that all metals are assumed to bond together if two cleaned surfaces are brought into contact within the range of interatomic force. Therefore, the key to realize diffusion bonding of aluminum alloy is to remove, or at least break, the continuity of oxide film during the bonding process [11]. In order to obtain a high quality bond joints of the aluminum alloy, many methods have been attempted to improve the bond quality of Al alloy or Al–Li alloy [12,13,14,15,16,17], for example: (1) diffusion bonding with a large pressure which can cause macroscopic plastic deformation to break the continuous oxide film; (2) transient liquid phase diffusion bonding with Zn or Cu as interlayer, this method needs a longer time to obtain a uniform bond joint; (3) the liquid Ga in room temperature to remove the surface oxide film and fulfill the diffusion bonding process, while the Ga caused liquid metal brittlement (LMB) of the component in the following service condition; (4) solid-state diffusion bonding assisted with Zn or Cu while the intermetallic formed and caused brittle fracture. According to our precious studies [18,19], the effect of active alloying element such as magnesium during diffusion bonding of aluminum-based alloys has been investigated, the results show that the alloying elements diffusion and the interfacial reaction between Mg and Al_2_O_3_ are beneficial to the bonding quality.

Based on the previous study above, from the point of view to improve the interface bond quality, the pure Al interlayer with excellent plasticity and an alloying element concentration gradient between the 1420 Al–Li alloy substrate was chosen to perform the diffusion bonding experiment. In our previous study, the active alloying element Mg can react with the oxide film and modify its characteristic and improve the bond quality effectively, the diffusion bonding of 1420 Al–Li alloy assisted by the pure Al foil as an interlayer was conducted under different parameters, the interface microstructure and mechanical property of the joints were investigated in detail.

## 2. Materials and Methods

**Figure 1:**
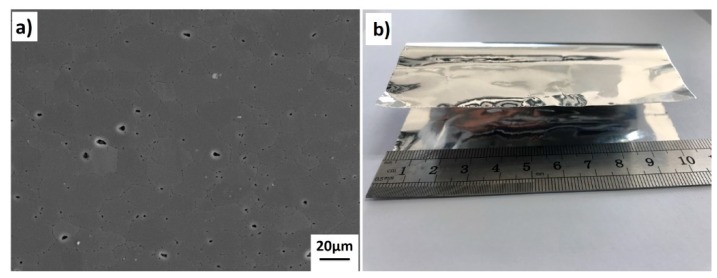
Microstructure of as-received 1420 Al–Li alloy plates (**a**) and image of pure Al foils (**b**).

The materials used in the present study was 1420 Al–Li alloy supplied by the Central South University, Hunan, China, country, the chemical composition of the as-received plates was detected by the inductively coupled plasma (ICP-Thermo iCAP 7400, Thermo Fisher Scientific, Massachusetts, MA, USA) and the results were shown in Table 1. Material was supplied in the form of plate with thickness of 2.2 mm, and has a density of 2.47 g/cm^3^. The microstructure of the as-received 1420 Al–Li alloy plate was shown in Figure 1a. The plates were supplied after a complex thermos-chemically treated process, it can be seen that the microstructure of 1420 Al–Li alloy primarily consists equiaxed grains which means the plate with superplastic properties. The pure Al foil with a thickness of 20 μm, as shown in Figure 1b, and the mass fraction of Al is no less than 99.999%.

Diffusion bonding was carried out on the Gleeble-3500 thermal simulation testing machine (Dynamic Systems Inc., Poestenkill, NY, USA) under the temperatures of 430–550 °C with a fixed pressure of 6 MPa for a time of 60 min. The temperature was measured by a K-thermocouple which fixed on the plate by resistance spot welding. The 1420 Al–Li alloy pre-bonding samples were processed by wire-cut electrical discharge machining with dimension of 2 mm × 10 mm × 15 mm. The bonding surfaces were ground using SiC abrasive paper and polished to a 1.0 μm diamond suspension. Pure Al foil with a thickness of 20 μm was applied as an interlayer to assist bonding of 1420 Al–Li alloy. Prior to diffusion bonding, the samples and foils were cleaned in an ultrasonic bath with acetone for approximately 5 min. Al foil was sandwiched between the 1420 base plates, then the diffusion bonding couples was fixed in the furnace. The diffusion bonding couple was heated to bonding temperature with a heating rate of 60 °C·min^−1^ and held at the objective temperature for 1 min to minimize temperature gradient. The bonding pressure was loaded on the bond couples during the entire bonding process. The main diffusion bonding parameters are listed in Table 2. Parameters such as bonding temperature, pressure and time were automatically controlled during bonding. In order to protect the samples from oxidizing, the work chamber was evacuated, until a pressure of 1.0 × 10^−3^ Pa was accomplished. After the bonding process, the pressure was released and the high pure argon was filled into the furnace to accelerate the samples cooling to room temperature.

**Figure 2:**
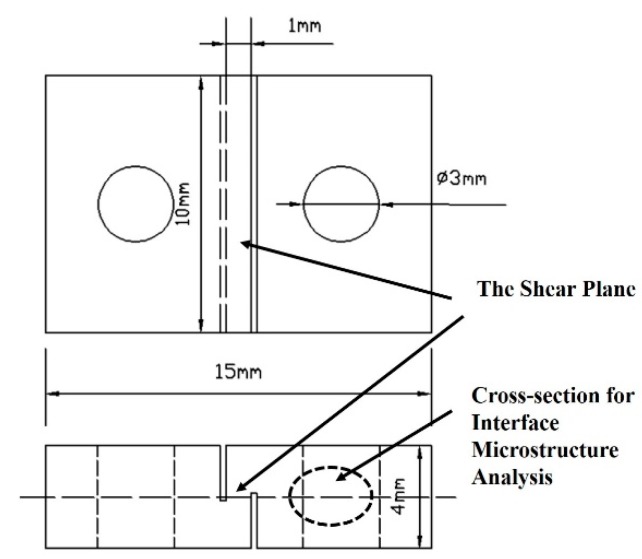
Schematic image of the tensile-shear strength test samples (unit: mm).

Mechanical properties of the joints were investigated through the nano-indentation hardness test and tensile–shear strength test under room temperature. Since diffusion bonding joints is at micro-scale, it is difficult to characterize the mechanical properties by the conventional hardness test method. With the rapid development technology of indentation and high precise surface image in recent years, nano-indentation gradually becomes an effective method to study the mechanical properties of materials at micro/nanometer scale. The micrographs of indentation and micro-nano mechanical properties of specific region can be obtained by the latest nanoindentation system, which has been an effective tool to study the local mechanical properties of materials at micro-nano scale, such as the hardness, residual stress, fracture toughness [20,21], etc. In the present study, the nano-indentation hardness was used to evaluate the effect of alloying element diffusion on the mechanical property of bond joint. The test was conducted on the MTS-NanoIndenter XP (MTS, Minneapolis, MN, USA), the polished specimen was fixed on the test bench of the nanoindenter. The test bench was moved to the proper position and then nanoindentation tests were carried out on the 1420 Al–Li alloy base materials and interlayer, respectively. The test parameters: the maximum load of penetration was 100 mN, Poisson’s ratio was set as 0.33, holding time in the maximum load was 10 s and then unloaded. The procedure of measurement can eliminate the influences of creep and thermal drift on the test results. Three sets of nanoindentation hardness of the diffusion bonding samples were measured across the bond joints at every 10 μm interval, the average value was taken to show the hardness change across the joints. The tensile-shear test combined with the fracture morphology were adopted to evaluate the bond strength and fracture mechanism of joints. The sample was processed for shear strength test after the bonding process, as the sample were not large enough for the standard tensile-shear test. According to the previous study [22,23], a non-standard tensile-shear test sample was devised as shown in Figure 2. The shear plane during the tensile-shear test and the cross-section for interface microstructure analysis was emphasized with the black arrows in Figure 2. The tensile-shear strength test was carried out on computer controlled electronic universal testing machine (WDW-50E, Lubiao test, Jinan, China) with a head speed of 0.3 mm·min^−1^, the initial tensile force was 0 N, and it increases as the tensile-shear process conducted, when the shear strength samples fractured under the tensile-shear force, the force was recorded as F_max_.

The shear strength value was calculated after the tensile-shear test, for each bonding parameter, three shear samples were tested and the average value was taken. The calculation formula of shear strength was shown as Equation (1)

τ_DB_ = F_max_/S, (1)

where τ_DB_ is the shear strength of bond joint, F_max_ is the maximum tensile force, S is the area of shear zone, in the present study, S is 10 mm^2^, as shown in Figure 2. The cross-section of joint were ground sequentially by 80 #, 600 #, 1000 #, 1500 # grit SiC paper, and then buff-polished with diamond pastes with a diameter of 1.0 μm, after which followed by etching with Keller’s reagent (90 mL H_2_O + 5 mL HNO_3_ + 3 mL HCl + 2 mL HF) for 15 s. A ZEISS SUPRA 55 scanning electron microscopy (SEM, ZEISS SUPRA 55, Oberkochen, Germany) with Oxford Aztec energy dispersive spectroscopy (EDS, ZEISS SUPRA 55, Oberkochen, Germany) was used to examine the interface characteristics and the alloying elements distribution across the bonding interface. In order to identify the phases in the diffusion zone, a Rigaku D/max-2550 X-ray diffractometer (XRD, Rigaku, Osaka, Japan) with Cu-K_α_ radiation (λ = 1.44184 Å) was used, the software used in the present study with the XRD equipment is DIFFRAC.EVA V4.1.1 and the MDI JADE, then the results were processed by the ORGIN 8.0. The morphology of fracture joint after tensile-shear strength test was observed with the SEM to study the failure mechanism.

## 3. Results

### 3.1. Interface Microstructure and Shear Strength

**Figure 3:**
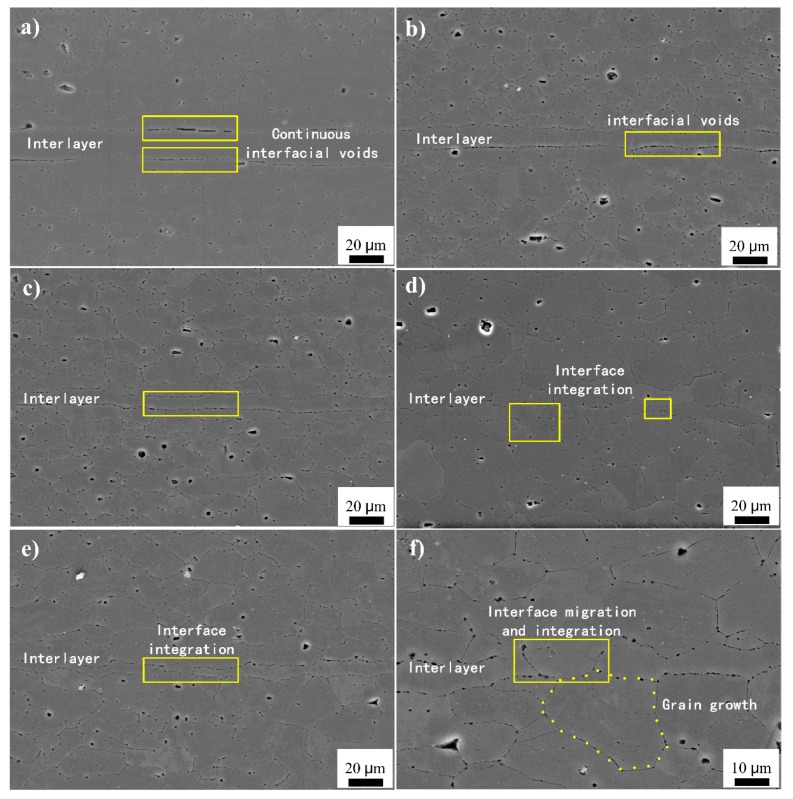
Interface microstructure of the joints obtained under different temperatures (6 MPa–60 min): (**a**) 430 °C; (**b**) 460 °C; (**c**) 490 °C; (**d**) 520 °C; (**e**,**f**) 550 °C.

The diffusion bonding of 1420 Al–Li alloy assisted by pure Al foil as interlayer were conducted under different bonding parameters, then the specimens were cut into shear samples followed by the tensile-shear test, after that the interface microstructure of joint was observed by the SEM to evaluate the interface quality. The interface microstructure and shear strength values of the joints bonded under different bonding temperature were shown in Figure 3 and Figure 5, respectively.

Figure 3 shows the interface microstructure bonded under different bonding temperature (6 MPa–60 min), the original interface is positioned in the center of image and indicated by the ‘interlayer’. It can be seen that the pure Al interlayer is different from the 1420 Al–Li alloy substrate, there are obvious plastic deformation of the pure Al interlayer during the bonding process due to its lower yield strength under bonding conditions, the thickness of the pure Al interlayer decreases with the increases of the bonding temperature. As shown in Figure 3a, when diffusion bonding was conducted at 430 °C (6 MPa–60 min), the thickness of the interlayer is around 20 μm, meanwhile only partial regions obtained metallic bond between the 1420 Al–Li alloy substrate and the Al interlayer, which show poor bonding quality. When the bonding temperature increased to 460 °C, the thickness of the pure Al interlayer decreased obviously, the bond regions increase evidently and only local unbonded areas distribute along the interface, as indicated by the arrows in Figure 3b. When the bonding temperature increased to 490 °C, there are no obvious unbonded regions distribute along the interface while only some small interfacial voids exist between the Al foil and the 1420 substrate. Figure 3d show the interface microstructure obtained under 520 °C, the thickness of the pure Al interlayer decreased obviously and the grains around the interface grow into the 1420 substrate. To further increase the bonding temperature to 550 °C, as shown in Figure 3e, no obvious interfacial voids can be found along the interface between the Al interlayer and 1420 Al–Li alloy substrate. Meanwhile, due to the interface migration caused by grain growth (as shown in Figure 3f), it is difficult to distinguish the original bonding interface from the grain boundaries in the 1420 Al–Li alloy substrate, which show good bond quality. The interface microstructure of the bond joints above is similar with the previous study by Pilling [24] about diffusion bonding of the 7475 Al alloy, which shows a good bond quality.

During the diffusion bonding, the shrinkage of interfacial void is an important process for achieving high quality bond joints because the following physical mechanisms [25,26,27]: (1) the plastic flow of materials around voids, including plastic deformation and creep deformation; (2) atomic diffusion, including surface diffusion, interface diffusion, and volume diffusion. In the present study, the increase of bonding temperature will reduce the yield strength of material, enhancing the plastic flow of materials, thereby forcing more mass from adjacent regions into voids. Meanwhile, the shrinkage of the interfacial voids is further accelerated by increased atomic diffusion.

**Figure 4:**
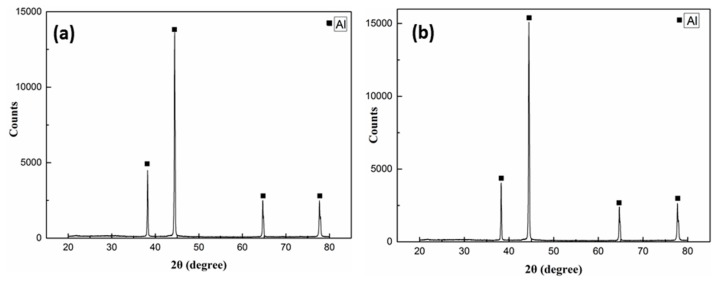
XRD result of the as-received plate (**a**) and diffusion bonding zone (520 °C–6 MPa–60 min) of 1420 Al–Li alloy assisted by pure Al interlayer (**b**).

Phase formation or transformation during the diffusion bonding process was confirmed by X-ray diffraction analysis. The XRD analysis of the as-received 1420 Al–Li alloy plate and the joint zone after diffusion bonding under 520 °C was shown in Figure 4, as the thickness of diffusion bonding joints is 4 mm while the thickness of pure Al interlayer is less than 10 μm (520 °C–6 MPa–60 min), hence influence of the Al interlayer on the XRD results is weak, compared with the diffraction peak of the as-received plates, only slightly change occurs the intensity of the diffraction peak after the bonding process, and no new diffraction peak appears. The results show that there are no interfacial phases formation or transformation during the bonding process, this is consistent with the results of interface microstructure observation in Figure 3.

**Figure 5:**
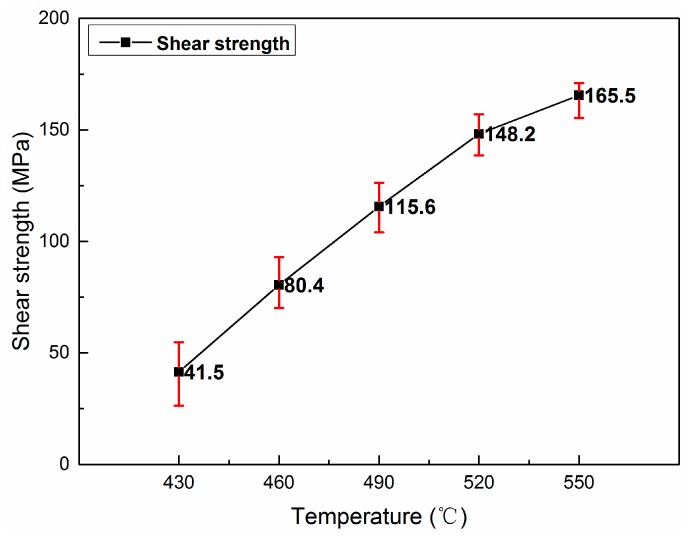
Shear strength of the joints which bonded under different temperatures (6 MPa–60 min).

The shear strength of bond joint is an important mechanical property to assess the bond quality. Figure 5 shows the effect of bonding temperature on the shear strength of the Al interlayer assisted diffusion bonding of 1420 Al–Li alloy. The results show the shear strength of the joints increase obviously with the bonding temperature. When diffusion bonding was conducted at 430 °C and 460 °C (6 MPa–60 min), the shear strength was only 41.5 MPa and 82.4 MPa, much lower than that of the as-received 1420 Al–Li alloy substrate. The shear strength of the joints increase to 115.6 MPa when the bonding conducted at 490 °C. To further increase the bonding temperature to 520 °C and 550 °C, the shear strength values can be as high as 148.2 MPa and 165.5 MPa, respectively.

### 3.2. Distribution of Mg and the Nano Indentation Hardness

**Figure 6:**
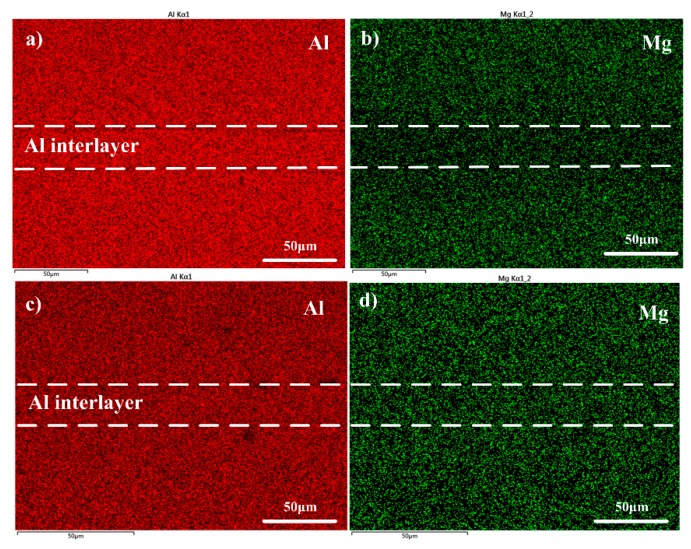
Main alloying element distribution across the bond joint: (**a**,**b**):460 °C–6 MPa–60 min; (**c**,**d**): 520 °C–6 MPa–60 min.

The alloy elements diffusion has direct effect on the bonding process. As the Li atom was too light to be detected by the EDS, the main alloying element Mg was chosen to investigate the alloying element diffusion, diffusion behavior, and its influence on the bond quality during bonding in detail. The distribution of the Mg across the joints obtained under different temperature is shown in Figure 6.

It can be seen from Figure 6a that there are obvious concentration gradient of Mg exists across the joints after diffusion bonding was conducted under 460 °C–6 MPa–60 min, which indicates in-homogeneous distribution of Mg across the bond joint. This is consistent with the interface microstructure in Figure 3b, which shows obvious interfacial defects and resulted in poor bond strength (as shown in Figure 5). To further increase the bonding temperature to 520 °C (6 MPa–60 min), the concentration gradient across the joints disappeared and homogeneous distribution of Mg was obtained, as shown in Figure 6d.

The results above can be ascribed to the different chemical composition between the 1420 Al–Li alloy and the pure Al interlayer which resulted in alloying elements diffusion across the interface. The alloying elements diffusion spontaneously from the 1420 Al–Li alloy substrate to the pure Al foil according to the Fick’s first law as shown in Equation (2).

J = −D·dC/dx,(2)

where J is diffusion flux (mol/(m^2^·s)); D is diffusion coefficient (m^2^·s^−1^); dC/dx is concentration gradient. Meanwhile, based on the Arrhenius-type equation (Equation (3))

D = D_0_exp(−Q/RT), (3)

where D is diffusion coefficient (m^2^·s^−1^); D_0_ is the proportionality constant (m^2^·s^−1^), independent of temperature for Equation (3) is valid; Q is the activation energy (J·mol^−1^); R is the molar gas constant (8.314 J·mol^−1^·°C^−1^); T is the temperature (°C). According to the above Equations (2) and (3), the diffusion flux across the bonding interface increase under the combined effect of temperature and alloying element concentration gradient, which can result in accelerating the shrinkage of interfacial voids and improve the bond quality.

**Figure 7:**
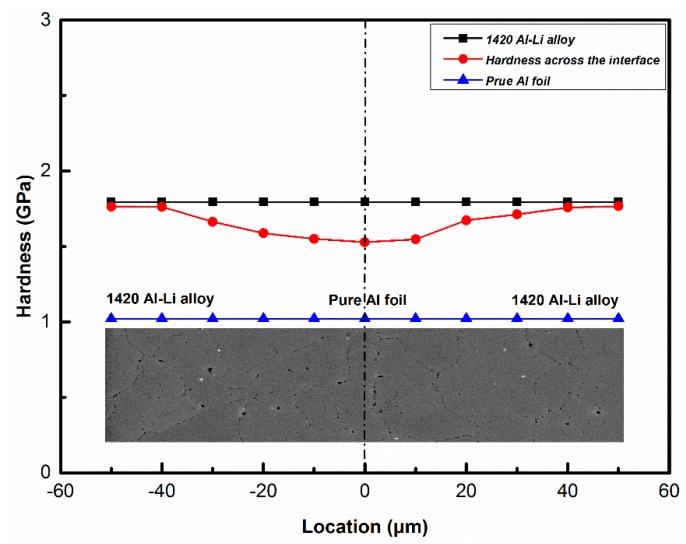
Nano-indention hardness distribution across the bond joint (520 °C–6 MPa–60 min).

The alloying elements diffusion can not only increase the diffusion fluxes across the interface, but also change the mechanical properties of the bonded area. The nanoindention hardness of the as-received pure Al foil and 1420 Al–Li alloy substrate are 1.021 GPa and 1.795 GPa, respectively. The nanoindention hardness test result across the bond joints was shown in Figure 7. It can be seen from the hardness of the interlayer increased obviously after the diffusion bonding process, the hardness value can be as high as 1.528 GPa, that because the alloying element such as Li and Mg diffusion into the pure Al interlayer which act as strengthen element and result in the increase of hardness. Meanwhile, an obvious hardness gradient formed around the joint areas, the hardness of 1420 Al–Li alloy substrate around the joints decrease can be ascribed to the loss of strength element during the bond process. This is similar with a previous study about the 8090 Al–Li alloy [28,29,30], the hardness of the surface layer decrease because the loss of strengthen alloy element. The results show that the alloying element diffusion can not only increase the interface integrity but also influence the mechanical property around the joint areas.

### 3.3. Shear Fracture Morphology

**Figure 8:**
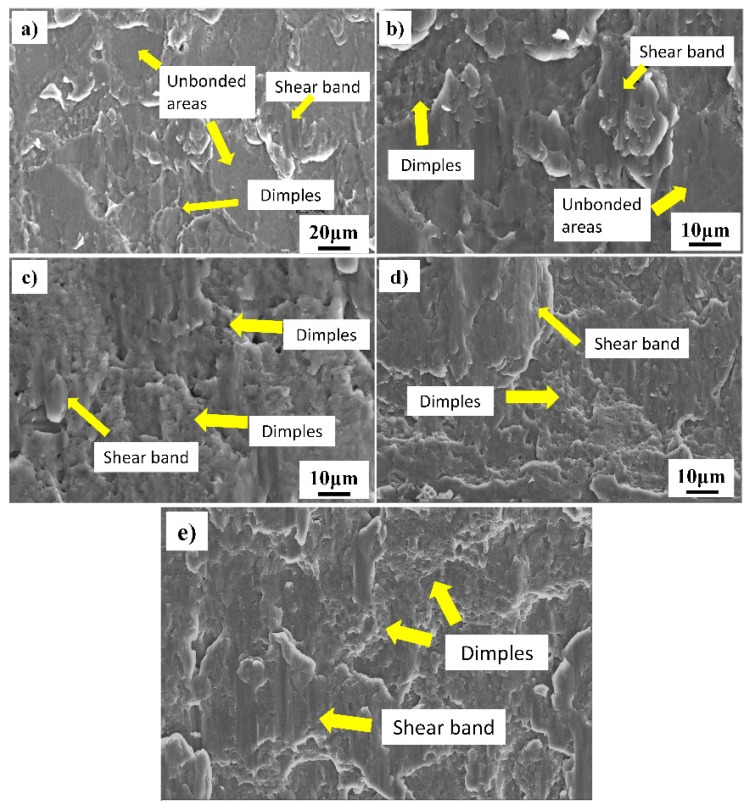
Shear fracture morphology of the joints bonded under different temperatures (6 MPa–60 min): (**a**) 430 °C; (**b**) 460 °C; (**c**) 490 °C; (**d**) 520 °C; (**e**) 550 °C.

After the shear strength test, the fracture surface was observed by SEM to evaluate the fracture mechanism. The fracture morphology of the pure Al interlayer assisted diffusion bonding of 1420 Al–Li alloys are shown in Figure 8. The results show that the fracture morphology corresponding to the interface quality in Figure 3, the interfacial voids in Figure 3 corresponding to the flat areas in Figure 8, these interfacial voids are likely to be the source of fracture along the interface when the joint is under external load, apparently this is not an effective bonding. The metallic bond areas corresponding to the shear band zones and dimples at the tip of the shear band. The fracture morphology shows that the increase of the shear band zones and dimples means better bond quality with higher shear strength. The dimples formed at the initial shear process and show ductile fracture characteristics, while the shear bands formed when the rapid fracture occurred at the last stage of the shear test. The results show that the shear fracture is a mixed fracture mechanism dominated by ductile fracture.

## 4. Discussion

### 4.1. Bond Formation Mechanism Assisted with Pure Al Interlayer

**Figure 9:**
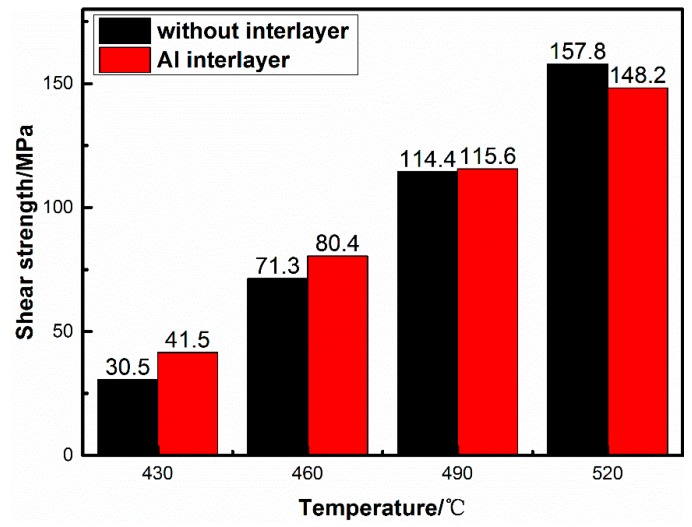
Comparison of shear strength with or without interlayer under different temperatures (6 MPa–60 min).

The interface integrity is usually used to assess the interface quality of the diffusion bonding joints. In the present study, from the hypothesis of high plastic flow and alloying element diffusion with the interlayer, the pure Al foil was chosen as interlayer in order to improve the bond quality. Compared with our previous study of the diffusion bonding of 1420 Al–Li alloy without interlayer [19], when diffusion bonding was conducted under 6 MPa-60 min for different temperature (460 °C /490 °C/520 °C), a higher interface integrity of bond joints obtained which can be ascribed to the plastic flow and alloying element diffusion. Meanwhile, according to the analysis of interface microstructure, XRD results and the distribution of the alloying elements across the bonded interface, there is no interfacial phases formation or transformation during the diffusion bonding process. Nevertheless, the shear strength is less than the bond joint of 1420/1420 when diffusion bonding was conducted at 520 °C–6 MPa–60 min (as shown in Figure 9), which can be ascribed to the poor strength of the pure Al interlayer. Although the mechanical property of the pure Al interlayer has been strengthened through the alloying element diffusion to some extent, but it still acts as the weakness zone in the shear strength test process.

Temperature is a key parameter which can directly influence the interface quality of bond joint. In the present study, the heat input (through the bond temperature) improved the formability of the 1420 Al–Li alloy substrate and the pure Al foil and promoted alloying element inter-diffusion at the interface of the joint. This can be concluded from Figure 3 and Figure 6, when the bonding temperature increased, the thickness of the pure Al interlayer decreased obviously and the distribution of Mg across the bond joints become more and more uniformly.

### 4.2. Prospects

Through the investigation on the diffusion bonding of 1420 Al–Li alloy in this study and our previous study, a sound bond joint can be obtained through the pure Al interlayer assisted diffusion bonding of 1420 Al–Li alloy and the diffusion bonding of 1420 Al–Li alloy and 7B04 Al alloy, the alloying element diffusion under concentration gradient can increase the diffusion fluxes of alloying element and result in the improvement of the interface integrity. Meanwhile, the interfacial reaction between the active alloying element and interfacial oxide film during the diffusion bonding process can modify the characteristic of oxide film and reduce its impediment on the diffusion, resulting in the improvement of the bond quality. The interlayer has different composition with the substrate can make good use of the alloying element diffusion and interfacial reaction; however, the pure Al foil has obvious different mechanical property with the substrate which can affect the bond strength.

Therefore, the key to improve the diffusion bond quality of 1420 Al–Li alloy is to choose the aluminum alloy as interlayer, on one hand, there is alloying elements concentration difference between the 1420 Al–Li alloy substrate, on the other hand, the mechanical properties of the interlayer is conducive to raise the bond strength of the joints, with the help of aluminum alloy interlayer, the diffusion bonding joints not only ensure the microstructure stability, but also have certain bond strength, this is also the focus in our future work.

## Figures and Tables

**Table 1:** Chemical composition of the as-received 1420 Al–Li plates (wt %)

Sample	Mg	Li	Zr	Cu	Fe	Si	Al
1420 Al–Li alloy	5.2	2.0	0.12	0.03	0.09	0.03	Bal.

**Table 2:** Diffusion bonding parameters in the present study.

Sample	Temperature (°C)	Presssure (MPa)	Time (min)
S1	430	6	60
S2	460	6	60
S3	490	6	60
S4	520	6	60
S5	550	6	60
